# Neurosensory Differentiation and Innervation Patterning in the Human Fetal Vestibular End Organs between the Gestational Weeks 8–12

**DOI:** 10.3389/fnana.2016.00111

**Published:** 2016-11-15

**Authors:** Lejo Johnson Chacko, Elisabeth J. Pechriggl, Helga Fritsch, Helge Rask-Andersen, Michael J. F. Blumer, Anneliese Schrott-Fischer, Rudolf Glueckert

**Affiliations:** ^1^Department of Otolaryngology, Medical University of InnsbruckInnsbruck, Austria; ^2^Department of Anatomy, Histology, and Embryology, Division of Clinical and Functional Anatomy, Medical University of InnsbruckInnsbruck, Austria; ^3^Department of Otolaryngology, Uppsala University HospitalUppsala, Sweden; ^4^University Clinics Innsbruck, Tirol KlinikenInnsbruck, Austria

**Keywords:** inner ear, vestibulum, human, transcription factors, PAX2, PAX6, PAX8, MAF B

## Abstract

Balance orientation depends on the precise operation of the vestibular end organs and the vestibular ganglion neurons. Previous research on the assemblage of the neuronal network in the developing fetal vestibular organ has been limited to data from animal models. Insights into the molecular expression profiles and signaling moieties involved in embryological development of the human fetal inner ear have been limited. We present an investigation of the cells of the vestibular end organs with specific focus on the hair cell differentiation and innervation pattern using an uninterrupted series of unique specimens from gestational weeks 8–12. Nerve fibers positive for peripherin innervate the entire fetal crista and utricle. While in rodents only the peripheral regions of the cristae and the extra-striolar region of the statolithic organs are stained. At week 9, transcription factors PAX2 and PAX8 were observed in the hair cells whereas PAX6 was observed for the first time among the supporting cells of the cristae and the satellite glial cells of the vestibular ganglia. Glutamine synthetase, a regulator of the neurotransmitter glutamate, is strongly expressed among satellite glia cells, transitional zones of the utricle and supporting cells in the sensory epithelium. At gestational week 11, electron microscopic examination reveals bouton contacts at hair cells and first signs of the formation of a protocalyx at type I hair cells. Our study provides first-hand insight into the fetal development of the vestibular end organs as well as their pattern of innervation by means of immunohistochemical and EM techniques, with the aim of contributing toward our understanding of balance development.

## Introduction

The human inner ear consists of both the vestibular and cochlear parts that form organs for balance and hearing. They start to develop during embryogenesis from the primordial surface ectoderm into the membranous labyrinth during the third gestational week (O'Rahilly, 1963). This surface ectoderm differentiates into the otic placode, otic primordium, and finally the otic vesicle. The otic vesicle finally separates into the primordium of the endolymphatic duct system, which consists of the vestibular portion, the cochlea, and the endolymphatic sac (Arnold and Lang, 2001). The membranous labyrinth in the inner ear contains a secluded epithelial layer that is diversified into specific regions containing sensory elements of the hair cells and supporting cells, and transmits information to local neurons ensheathed by Schwann cells and satellite glia. All the cells of the inner ear are derived from the otic placode with the exception of the Schwann cells (Alsina et al., 2009), satellite glia, strial intermediate cells (neural crest), and potential cells belonging to the reticulo-endothelial system (RES). The embryogenic developmental studies of the human inner ear, especially the vestibular organ, are hindered by the lack of insight regarding the developmental consequences (neural tube signals/cell fate determination) of the activation and inactivation of specific genes which have been linked to development of the inner ear in murine embryogenic models. Also, there is a lack of comparative studies regarding the activation of these specific marker genes and their influence during the development of the vestibular organ and the cochlea. Here, we used different molecular markers to characterize sensorineural progression from gestational week 8–12 in human embryogenesis.

### Innervation-peripherin and class III β-tubulin

Peripherin is a type III neuronal intermediate filament protein found to selectively stain for the type II SGNs of the adult human cochlea (Liu et al., 2010; Barclay et al., 2011). It has also been found in the sensory neurons of the peripheral nervous system (PNS) and is widely expressed during rat embryogenesis (Escurat et al., 1990). Studies carried out on mice embryonic models have shown the presence of peripherin in the ninth day embryonic of development, and the pattern of staining indicates that peripherin staining occurs similarly during embryogenesis in adult tissues (Troy et al., 1990). Parallel studies carried out on rat embryonic models also demonstrated positive staining for peripherin at the eleventh day of embryonic development (Gorham et al., 1990).

Class III β-tubulin is a microtubule element connected to neuronal differentiation in the Central Nervous System (CNS; Katsetos et al., 2003) and presents mostly in neurons of the mature and regenerating hair cells of the inner ear in avian (Molea et al., 1999) and mammalian species. Similar analysis performed on human tissue has also shown the expression of the class III β-tubulin across the spiral ganglion neurons (SGN) in the fetal (Pechriggl et al., 2015) and adult cochlea (Barclay et al., 2011). It has also been revealed that the β III tubulin isotype is found not only in the soma, axon and the bouton type terminal dendrite compartments, but also in the big calyceal nerve endings (Perry et al., 2003). Staining intensity for this microtubule element was found to increase from week 8–12 of embryonic development in the human fetal cochlea (Pechriggl et al., 2015) and may be necessary to establish axonal function.

### Neurotransmission associated proteins-synaptophysin and glutamine synthetase

Synaptophysin is a synaptic vesicle glycoprotein distributed across the peripheral vestibular afferent neurons in the developing mouse embryo (Scarfone et al., 1991) as well in the vestibular nerve calyx that ensheathes the type I inner sensory hair cells of the vestibular epithelium (Scarfone et al., 1988). It is the most abundant integral membrane protein of small presynaptic vesicles in the human cochlea (Khalifa et al., 2003). Other studies performed during late postnatal development in mice have also shown that synaptophysin staining occurs in the nerve terminals of efferent neurons in the vestibular end organs (Dechesne et al., 1997). Synaptophysin expression indicates the initiation of the excitation process in the nerve fibers of the developing chick inner ear (Sokolowski and Cunningham, 1996). Expression of the same has also been linked to the release of neurotransmitters (Sokolowski and Cunningham, 1999). Studies performed on rat embryo models have shown that synaptophysin is restricted to efferent neurons and is down-regulated in these neurons indicating that synaptogenesis of efferent fibers is complete (Knipper et al., 1995).

Glutamate is the neurotransmitter in the hair cells of the inner ear (Nordang et al., 2000), hair cells that are not surrounded by glial cells like they are in the CNS (Puel, 1995). Acoustic overstimulation in glutamate aspartate transporter (GLAST)-deficient mice was found to cause an accumulation of glutamate in the perilymph leading to a deterioration of hearing loss, lending credence to the premise that glutamate is indeed the neurotransmitter for hair cells (Hakuba et al., 2000). Degradation of synaptically released glutamate is thus important for normal function. GLAST and glutamine synthetase that metabolizes glutamate to glutamine, respectively, may operate in concert to terminate the neurotransmitter action of glutamate (Miller et al., 2002). In adults, glutamine synthetase is located in the satellite glia cells surrounding many ganglion neurons (Hanani et al., 2005). Immunohistochemical analysis of human fetal cochlear specimens has shown that the expression of glutamine synthetase-positive peripheral glial cells starts as early as the eighth week of gestation (Pechriggl et al., 2015).

Non-sensory cells—S100 proteins are normally present in cells derived from the neural crest in the inner ear Schwann cells, chondrocytes, melanocytes, and intermediate cells of the stria vascularis. During development, a variety of other cells including ganglia and hair cells (Buckiova and Syka, 2009) express S100 isotypes that do not overlap in expression with other calcium binding proteins. Studies performed on embryonic mice models have shown the expression of the calcium binding protein S100, which can locally regulate neuronal plasticity (Kiryushko et al., 2006) in the inner ear beginning on embryonic day 14 onwards, in both the vestibular and cochlear epithelial cells. This pattern of staining then progresses into the end organs of the vestibulum by embryonic day 17.

### Cellular patterning-MAF B and the PAX gene family

The downregulation of the transcription factor Maf B (V-maf musculoaponeurotic fibrosarcoma oncogene homolog B), which is encoded by the MAF B gene in humans, has been found to affect the kr/Maf B-hindbrain pathway progression in fetal murine models, which in turn leads to a downregulation of the otic genes involved in normal inner ear cell patterning like the Gbx2, Dlx5, Wnt2b, and Otx2. This deviation in the expression pattern in the dorsal region of the developing otocyst leads to the loss of dorsal otic structures such as the endolymphatic duct and the sac (Choo et al., 2006). Studies performed on a MAF B KO model have also confirmed the abnormal inner ear development (Terrell, 2006). Immunohistochemical analyses of human fetal cochlear specimens have shown that the expression of this transcription factor is seen as early as the eighth week of gestation and helps to distinguish cochlear neurons from other cells in the spiral ganglion (Pechriggl et al., 2015).

Pax2 is a protein which encodes a transcription factor necessary for the development of the cochlea in mice as well in humans. Moreover, inactivation of Pax2 has been found to cause varying degrees of vestibular defects as well as an associated loss of sensory neurons. Studies performed using Pax2;8 double null mice has shown that there is incomplete neuronal as well as neurosensory development in the inner ear and that Pax2 expression compensates for Pax8 in a Pax8 null mouse model (Bouchard et al., 2010). Pax2 expression has been found at varying intensities in some vestibular regions in a developing chicken inner ear model (Sánchez-Calderon et al., 2002), while loss of function experiments using the same model have shown the necessity of Pax2 for the development of the cochlear duct as well as its presence in the sensory and non-sensory structures in the inner ear (Sánchez-Calderón et al., 2005). Knockout as well as frameshift mutant Pax2 murine models revealed agenesis or severe deformity of the cochlea. Pax2 mutations have been found to cause mild sensorineural deafness in humans (Schimmenti et al., 1997). Other studies performed using embryonic chick models have also suggested a coordinating but independent role for Pax2 in epithelial morphogenesis and cell fate determination during inner ear development (Christophorou et al., 2010). However, in studies performed using a Pax2 null mice model, the vestibular sensory cells of the inner ear were found to develop normally and establish their innervation with their associated vestibular ganglion (Torres et al., 1996). We found Pax2 expression previously in the fetal human cochlea in the SGNs as well as in the inner hair cells (IHCs) indicating its role in the early maturation of the IHCs in comparison to the outer hair cells (OHC; Pechriggl et al., 2015).

The Pax6 gene encodes the transcriptional factor, Paired Box Protein Pax-6 which is involved in sense organ patterning and forebrain development (Georgala et al., 2011) with its expression *not being previously described in the fetal human inner ear*.

Research performed using embryonic Xenopus models have identified Pax8 as the most primitive indicator of the development of the otic placode and of the intermediate mesoderm. Moreover, it has also been suggested that Pax8 plays a central role in the auditory development in the same model (Heller and Brändli, 1999). Additionally, findings from Zebrafish models have indicated that Pax8 is also the earliest developmental marker for the otic placode and that inactivation of Pax2 causes the development of the otic region to halt, thereby indicating that Pax8 expression is very much dependent on the expression of Pax2 (Pfeffer et al., 1998). Though, Pax8 expression has been connected to the induction of the otic placode and its further development, null Pax8 mice models have been found to have a normally developed inner ear (Mansouri et al., 1998). Normal development of the inner ear, even in the absence of Pax8, has been suggested to be due to the redundant function either of an unrelated gene or of another member of the Pax gene family (Chatterjee et al., 2010). From studies carried out on null mice models of both Pax2 and Pax8, this redundant functionality was later identified to be due to the ability of Pax2 to overcome the non-functionality of the Pax8 (Bouchard et al., 2010). Moreover, 3D stem cell models of ear sensory epithelia have shown the upregulation of both Pax2 and Pax8 during development of the otic placode (Koehler et al., 2013).

Proliferation—Ki-67 is a nuclear protein involved in ribosomal RNA transcription and is involved in cellular proliferation. Ki-67 is evenly distributed throughout the otocyst development and decreases as the embryonic development progresses, becoming spatially restricted within the membranous labyrinth epithelium (Tafra et al., 2014). Embryonic human tissue of equal gestational stages has revealed staining for Ki-67 in the spiral ganglion region as well as in the epithelium of the cochlear duct in the developing cochlea (Pechriggl et al., 2015).

Programmed cell death or apoptosis occurs widely during development. Caspases actively regulate animal development through both apoptosis and non-apoptotic functions that involve cell–cell communication in developing cell communities (Miura, 2011). The absence of the downstream apoptotic enzyme Caspase-3 has been found to induce hearing loss as well as vestibular dysfunction in a Caspase-3 deficient mouse model (Makishima et al., 2011). In our previous study on the human fetal cochlea, we did not find any clue for an involvement of this enzyme during development (Pechriggl et al., 2015).

We hypothesize that humans and rodents display different patterns of inner ear development characterized by differing patterns of gene expression. The goal of this study is to present novel insights into the developmental changes occurring in the human fetal vestibular sensory organs orchestrated by various neuronal markers (beta III tubulin, peripherin, synaptophysin, and MAF B), non-sensory (S100), neurotransmitter related enzymes (glutamine synthetase), transcriptional factors (Pax2, Pax6, and Pax8), and markers for proliferation and apoptosis (Ki-67 and cleaved Caspase 3) which are of particular interest for cell fate and proliferation. Previous studies have engaged murine models where the onset of hearing is delayed to several days after birth (Kraus and Aulbach-Kraus, 1981) unlike in humans, making it difficult to draw inferences from animal developmental models and thus pertinent to mapping the expression patterns of these markers in the fetal inner ear. Moreover, we have used a combinatorial approach involving both immunohistochemistry and transmission electron microscopy to identify and track the developmental changes occurring in the fetal human inner ear.

## Materials and methods

### Embryonic, fetal specimens

Twenty four human embryos (4/W8; 4/W9; 4/W10; 6/W11; 6/W12) aged between 8 and 12 weeks after conception corresponding to the anatomical age were obtained from lawful abortions which were performed in concordance with Austrian legislation (§ 97 StGB) and with prior parental approval. These are the only developmental stages during which it is legal to acquire an embryo. These specimens exhibited no macroscopic malformations and their embryological ages were differentiated by quantification of the crown-rump length, external and internal morphology, and the estimated gynecological age. This research was approved by the ethics commission of the University Clinic for Gynaecology and Obstetrics at the Medical University of Innsbruck, ethical approval no. AN2014-0095 335/4.11.

### Tissue preparation for histology and immunohistochemistry on paraffin sections

Temporal bone specimens were excised and then promptly fixed by steeping in ice cold 4% paraformaldehyde (PFA) in phosphate buffered saline (PBS, 0.1 M) at a pH of 7.4 and left overnight. Subsequently, the specimens were rinsed in PBS and were then prepared by dehydrating and later embedded in paraffin using a histological infiltration processor (Miles Scientific Inc., Naperville, IL, USA). Sequential sections of 4 μm thickness were made on a HM 355S microtome (Microm, Walldorf, Germany) and affixed on SuperFrost® Plus slides (Menzel, Braunschweig, Germany). The affixed specimens were dried overnight at room temperature. Afterwards, the section-containing slides were incubated at 60°C for 2 h to adhere the sectioned specimens firmly onto them. Every tenth specimen of each complete series was stained with haematoxylin/eosin (HE) (Shandon Varistain 24-4, Histocom Vienna, Austria).

### Antisera

The hosts, dilutions, and sources of primary antibodies used are listed in Table 1.

**Table 1:** **Antibodies used in this study**.

**Ab clone**	**Staining pattern**	**Host description**	**Background**	**Ab dilution pre-treatment**	**Supplier cat. no**.
Beta III tubulin (TUBB3)	Cytoplasmic	Rabbit polyclonal	β-Molecule of microtubules-neuron specific marker (Molea et al., 1999)	1:1500 CC1-standard	Abcam Cambridge, UK ab 18207
Peripherin (PRPH) 3B3	Cytoplasmic	Mouse monoclonal	Type III neuronal intermediate filament (Liu et al., 2010; Barclay et al., 2011)	1:400 CC1-standard	Lifespan Seattle, WA, USA LS-B6138
MAF B polyclonal	Nuclear	Rabbit polyclonal	Basic leucine-zipper containing transcription factor family; transcription factor that is expressed in (Choo et al., 2006; Marrs and Spirou, 2012)	1:600 CC1-standard	Sigma-Aldrich St. Louis, MO, USA HPA005653
Synaptophysin YE269	Nuclear	Rabbit monoclonal	Presynaptic vesicle protein p38 in neuronal and endocrine cells (Khalifa et al., 2003)	1:600 CC1-standard	Abcam ab 32127
S 100 4C4.9 (Raised against ß-chain)	Cytoplasmic	Mouse monoclonal	Calcium binding protein (Liu and Rask-Andersen, 2014)	ready to use	Ventana Roche Mannheim, Germany 790-2914
PAX2	Nuclear	Rabbit polyclonal	Paired box gene 2 transcription factor (Fekete and Wu, 2002)	1:35 CC1 standard	Invitrogen, Camarillo, CA, USA 18-0483
PAX6	Nuclear	Rabbit polyclonal	Paired box gene 6 transcription factor (Xu et al., 1999)	1:18 CC1 standard	Sigma Life Sciences, St Louis, MO, USA HPA030775
PAX8	Nuclear	Mouse monoclonal	Paired box gene 8 transcription factor (Lotan et al., 2009)	ready to use	Ventana Roche Mannheim, Germany 760-4618
Ki-67 (30-9)	Nuclear	Rabbit monoclonal	Proliferative activity of normal and neoplastic tissue (Takebayashi et al., 2005)	ready to use CC1-standard	Ventana Roche 790-4286
Cleaved caspase 3 (Asp 175)	Nuclear	Rabbit polyclonal	Marker for apoptosis (Gown and Willingham, 2002)	1:400 CC1-standard	Cell Signalling Boston, MA, USA 9661
Glutamine synthetase polyclonal	Cytoplasmic	Rabbit polyclonal	Catalyses the production of glutamine and GABA (Eybalin et al., 1996; Procacci et al., 2008)	1:10000 CC1 standard	Sigma-Aldrich G2781

### Immunohistochemistry

Immunohistochemistry was rendered with a Ventana Roche® Discovery XT Immunostainer (Mannheim, Germany), using a DAB-MAP discovery research standard procedure. When necessary, antigen retrieval was commenced by unmasking the epitope using a heat induction method while the sections were immersed in EDTA buffer (Cell Conditioning Solution CC1, Ventana 950–124).

The mounted sections were incubated with the appropriate primary antibodies at 37°C for 1 h. Following this the specimens were incubated with Discovery Universal Secondary Antibody, Ventana 760–4250 at room temperature for 30 min. Antibody detection was attained with the DAB-MAP Detection Kit (Ventana 760–124) using a combinatorial approach involving the diaminobenzidine development method with copper enhancement followed by light counter staining with haematoxylin (Ventana 760–2021) for 4 min. The stained sections were then manually dehydrated using an upgraded alcohol series, clarified with xylene and then mounted permanently with Entellan® (Merck, Darmstadt, Germany).

The entire immunohistochemical staining reaction was benchmarked against appositive controls (e.g., small intestine, brain, and pancreas) that were supplemented to each experiment. Auxiliary negative controls were acquired by alternating the primary antibodies with reaction buffer or substituting them with isotype matching immunoglobulins. These ancillary controls never yielded any immunostaining.

### Image analysis of HE-staining and immunohistochemistry

The histological and immunostained sections were digitally amassed using a Zeiss AxioVision 4.1 microscope software coupled to an AxioCam HRc color camera and an AxioPlan2 microscope (Zeiss, Jena, Germany).

### Tissue preparation for light (LM) and transmission electron microscopy (TEM) analysis

Two fetal inner ear specimens (2/W11) were detached and divided mid-modularly and were fixed in 2.5% glutaraldehyde and 2% paraformaldehyde buffered in sodium cacodylate (0.1 M, pH = 7.4) over night at 4°C. Since the development of the principal fluid spaces in the fetal inner ear was not yet complete, this partitioning of the cochleae allowed for excellent fixation quality for ultrastructure analysis. Subsequently they were rinsed in sodium cacodylate buffer and post-fixed in 1% osmium tetroxide in distilled water for 3–4 h at 4°C. Again, samples were rinsed, dehydrated in graded ethanol series and embedded in EPON resin.

Ultrathin sections (90 nm) were cut on a Reichert Ultracut S microtome (Leica Microsystem, Wetzlar, Germany) with an ultra-diamond knife, mounted on dioxan-formvar coated slot-grids (#G2500C, Christine Gröpl, Elektronenmikroskopie, Tulln, Austria) and stained 35 min with 0.5% (w/v) uranyl acetate, pH 4.4 and 10 min with 3% (w/v) lead citrate, pH 12 (Leica Ultrostainer, Leica Microsystem, Wetzlar, Germany). The ultrathin sections were examined with a Philips CM 120 transmission electron microscope at 80 kV (FEI, Eindhoven, Netherlands) equipped with a MORADA digital camera (Olympus SIS, Münster, Germany).

## Results

### Sensory innervation and cell proliferation at W8 and W9

At W8 all sensory organs were clearly identified and contained hair cells as well as neighboring supporting cells. Semi-circular canals had already formed with the vestibular neurons sending out axons toward the peripheral as well as central processes.

**Figure 1:**
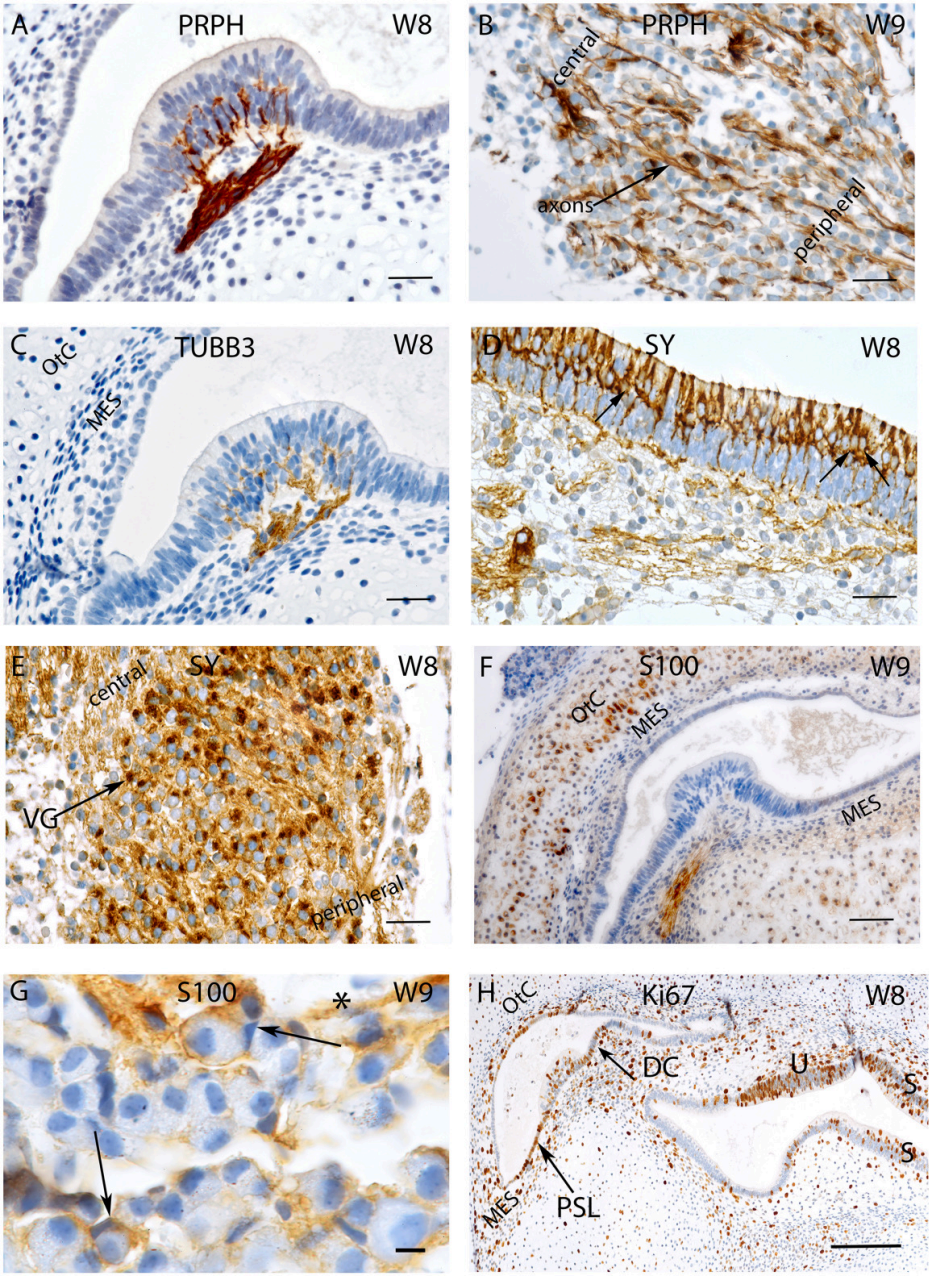
**Sections of the developing VO between W8 and W9. (A)** Peripherin staining in the Crista ampullaris (intensely stained Peripherin positive fibers are invading the sensory epithelium) and **(B)** the vestibular ganglion; peripheral as well as central axons of the vestibular nerves are immunoreactive. Fibers immunoreactive for TUBB3 are innervating the sensory epithelia of a crista ampullaris **(C)** by W8. The otic capsule (OtC) and the mesenchyme (MES) are all unstained. **(D)** Synaptophysin stained nerve fibers innervate the sensory epithelia of the developing utricule where immunoreactive hair cells and intensely stained nerve endings (arrows) are present. **(E)** Synaptophysin expression is also widespread in the vestibular ganglion (VG) cells with an apparent gradient in the staining intensity between the central and the peripheral regions of the ganglia. **(F)** S100 immunoreactivity is widespread in the ampullary crest with the nerve fibers projecting toward the sensory epithelia. The cytoplasm of the chondrocytes of the otic capsule (OtC) show immunoreactivity for S100. **(G)** S100 expression is also visible in the VG within stained putative Schwann cells (asterisk) and satellite glial cells (arrows) stained. **(H)** Ki-67 immunoreactivity is seen in few cells of the ampullary crest (H), within in the regions of the Planum semilunatum (PSL), dark cells (DC) of the cristae, sensory epithelium of the utricle (U), saccule (S) and the developing mesenchyme (MES). Scale bar: 12 μm **(A–F,H)**, 10 μm **(G)**.

The vestibular end organs exhibited intense staining for the type-III neuronal intermediate filament **peripherin** in the nerve fibers innervating them. These fibers appeared between supporting cells and basal portion of hair cells (Figure 1A). Peripheral as well as central processes of Scarpa ganglia display high intensity of peripherin signal. Only few neurons showed staining in their somata. Different staining intensities may be related to unequal development within the neuronal population (Figure 1B). Peripherin previously identified as a marker for type II spiral ganglion cells in adults constituted only 5–10% of the total ganglion cell population (Spoendlin, 1985). A similar characterization was not reported for vestibular ganglion cells.

Staining for β-**III tubulin** was confined to the nerve fibers projecting into the sensory epithelia (Figure 1C). Expression for this marker was present in the nerve fibers which traversed the mesenchyme and projected into the sensory epithelium of both ampullary as well as the otolithic organs—the utricle and the saccule at W8. Peripheral as well as central processes revealed staining but were less pronounced than peripherin (compare Figures 1A,C). Scarpa perikarya were only faintly stained or void of any immunoreactivity.

Expression was also seen for the synaptic vesicle glycoprotein—**synaptophysin**—which was found in the axons and vestibular hair cells (Figure 1D) as well as within the cytoplasm of all the neurons (Figure 1E). Expression for this protein was observed among the axons of the nerve fibers lying underneath and projected toward the vestibular sensory hair cells lining the utricule (Figure 1D). Axoplasmic distribution was clearly observed; however, this staining was not evenly distributed and was enriched in the nerve terminals underneath the hair cells (Figure 1D, arrows). The entire cytoplasm of hair cells was intensely stained and augmented at the cuticular plates, while stereocilia and cilia were void of any immunoreactivity (Figure 1D). Staining patterns parallel to that of the utricule were apparent in the ampullary crests and the saccule. Synaptophysin expression was also widespread in the Scarpa ganglion neurons and seemed to be concentrated closer to the bigger central axons (Figure 1E). Though stained nerve fibers where visible in the spiral ganglion of the cochlea, no penetration into the sensory epithelium was observed till week 12 of gestation in the cochlea (Pechriggl et al., 2015), reflecting the advanced development of the vestibular system.

The low molecular weight protein **S100** was widely distributed at W8 and stained the cytoplasm of most chondrocytes of the otic capsule as well as the mesenchymal tissue to a much lesser extent (Figure 1F). The most prominent immunoreactivity appeared along the nerve fibers lying within the stroma and projected toward the sensory epithelia (Figure 1F). The Scarpa ganglion perikarya were not stained by this antibody (Figure 1G), but many cells surrounding the ganglion were. Satellite glia cells that formed a thin sheet around some neurons showed reactivity. Putative, non-myelinating Schwann cells appeared stained (Figure 1G), and expression for S100 was very similar in the cochlea (Pechriggl et al., 2015).

The embryonic vestibular organs (VO) at W8 also exhibited staining for the nuclear protein **Ki-67**. Staining for this antibody was found among the cells of the planum semilunatum layer (PSL), dark cells as well as the transitional cells adjacent to the cristae (Figure 1H). This indicated that growth of the endolymphatic compartment was mostly contributed to by proliferation of these cells and by patches of the simple cuboidal epithelial lining of the membranous labyrinth. Staining was also seen in the mesenchymal cells lying underneath the cristae as well as in the chondrocytes that reflected the proliferation needed for the growth of the otic capsule.

### Glutamine synthetase and transcriptional regulator MAF-B

**Figure 2:**
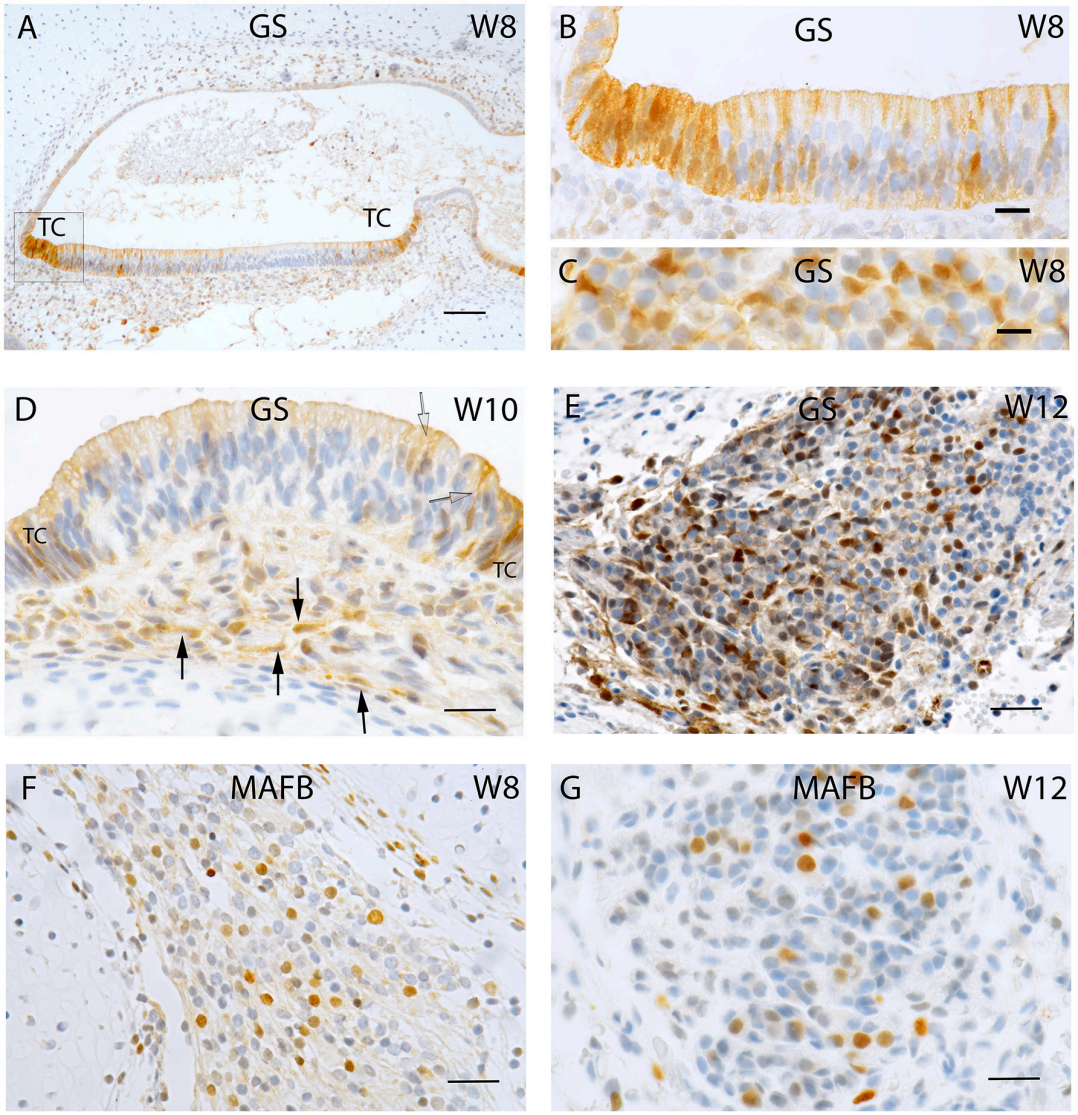
**Sections of the developing fetal VO between W8 and W12. (A)** Glutamine synthetase (GS) expression in the **(A)** Utricle at W8 in the transitional cells (TC) and the supporting cells of the otic epithelium. **(B)** Higher magnified view of the framed area in **(A)** of the TC and neighboring sensory epithelium where the supporting cells exhibit strong GS immunoreactivity while the hair cells are void of staining. **(C)** Higher power view of the VG from W8 were only the satellite glial cells shows strong GS immunoreactivity. **(D)** GS immunoreactivity in a crista ampullaris at W10 were the TC and supporting cells in the sensory epithelium (open arrow) are positive for this marker. Non-myelinating Schwann cells (arrows) under the ampullary organs also show immunoreactivity for this marker. **(E)** GS in the neurons at W12. Strong immunoreactivity is visible in the satellite glial cells at this stage. **(F)** MAF B immunoreactivity is present in a subpopulation of VG cells at W8. **(G)** MAF B again stains nuclei in a subpopulation of ganglion cells in a manner reminiscent of W8. Scale bar: 50 μm **(A)**, 10 μm **(B,C)**, and 12 μm **(D–G)**.

Expression for the enzyme glutamine synthetase necessary for “detoxification” and recycling of the neurotransmitter glutamate was strongly expressed among the transitional zones of the utricle and supporting cells in the sensory epithelium (Figures 2A,B). Within the ganglion, satellite glia cells appeared stained (Figure 2C), parallel to the adult situation in the spiral ganglion (Pechriggl et al., 2015). Interestingly, at week 10, the putative, non-myelinating Schwann cells showed distinct immunoreactivity underneath the ampullary organs. Here again, transitional zones as well as supporting cells were positive while the hair cells were negative (Figure 2D). In the vestibular ganglion, satellite glia cells were strongly stained at week 12 (Figure 2E).

Nuclear staining for MAF B was confined to a subpopulation of Scarpa neurons at week 8 (Figure 2F) as well as at week 12 (Figure 2G), indicating that neural differentiation was still ongoing.

### Sensory innervation and cell proliferation at W10 and W11

**Figure 3:**
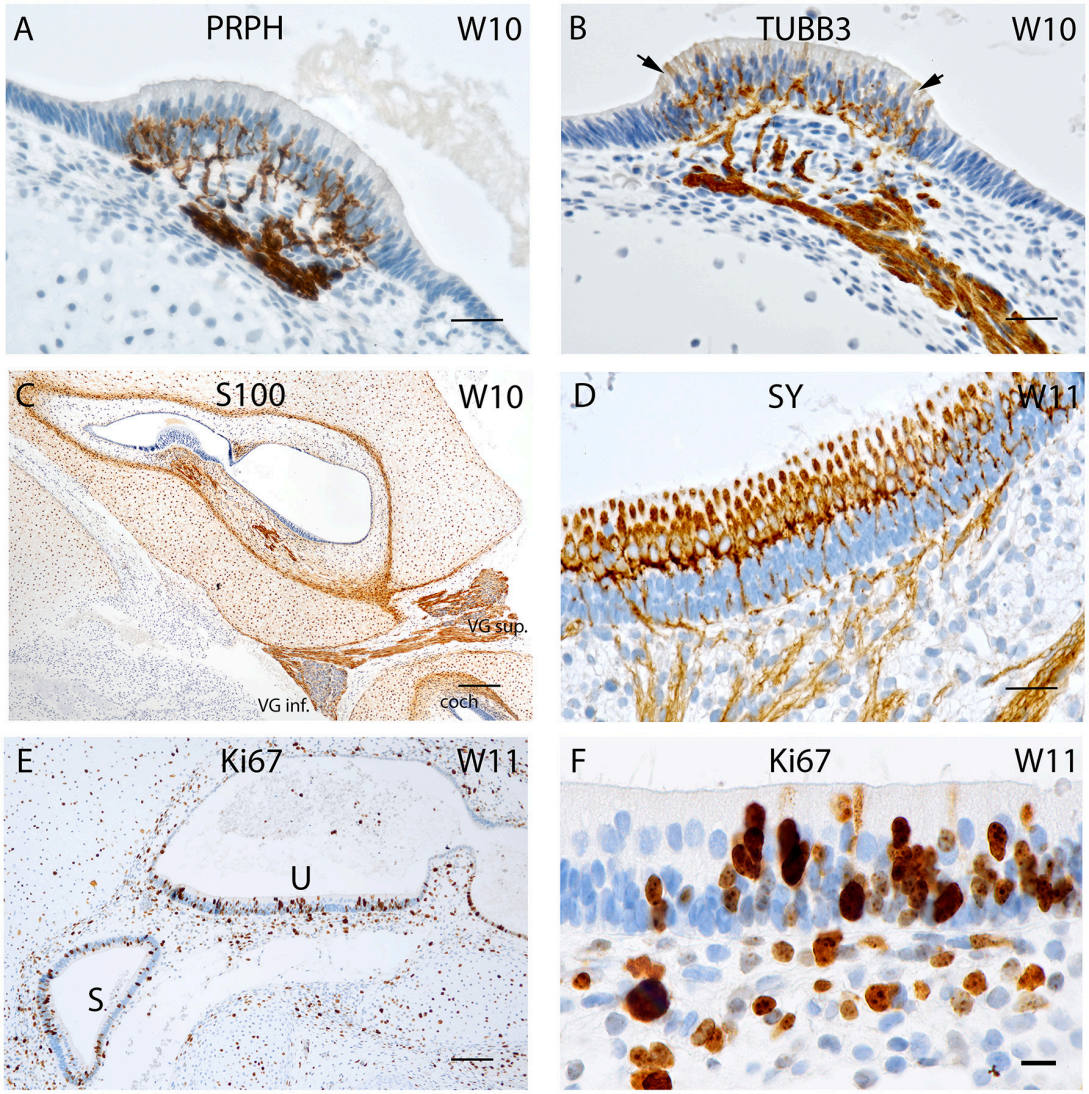
**(A)** Peripherin staining of the cristae ampullaris at W10. The staining pattern is similar to that seen in W8 with the positive fibers extruding into the basal portion of the sensory hair cells. **(B)** Beta III tubulin staining in a crista ampullaris. Strong immunoreactivity in the nerve fibers. **(C)** S100 staining is present in all cell types except neurons and otic epithelium lining the vestibulocochlear duct. At the transition between otic capsule and mesemchymal stroma pronounced immunoreactivity delineates the later borders of the bony labyrinth. **(D)** Synaptophysin immunoreactivity deepens at W11 in the nerve fibers reaching the sensory cells of the utricle where the hair cells express SY while the supporting cells do not. **(E)** Ki-67 immunostaining in the proliferating cells of the macular organs at W11 (U, utricle; S, saccule). **(F)** Ki-67 expression is visible in the basal cell layer of the utricular epithelia as well as in the intermediate and apical region of the epithelium that could be hair cells. Scale bar: 12 μm **(A,B,D)**, 10 μm **(F)**, 50 μm **(C,E)**.

**Peripherin** immunoreactivity and staining pattern at W10 equalled that of the previous weeks. Positive fibers reached the basal portion of the sensory hair cells in ampullary sensory patches (Figure 3A) as well as in the otolith sensory epithelium. In contrast, in the cochlea, only few stained fibers were to be found within the prosensory domain (Pechriggl et al., 2015), reflecting the advanced developmental time line of the vestibular system as compared to the auditory.

Intense immunolabelling was also observed for **Beta III tubulin** in the nerve fibers protruding and innervating the developing crista ampullaris (Figure 3B) as well as other vestibular sensory hair cells at W10. The utricle at this stage exhibited intense staining for this neuronal filament with the nerve fiber bundles stained and traversing through the mesenchyme and projecting into the sensory epithelia. Single, faintly stained apical portions of vestibular sensory hair cells were also visible at this stage with some putative nerve fibers overshooting the basal portion of hair cells (Figure 3B, arrows). We had previously observed a similar overshoot in the developing auditory system (Pechriggl et al., 2015).

Staining for the **S100** proteins weakened in the cytoplasm of chondrocytes but increased dramatically in the cells at the border between the cartilage and mesenchymal tissue, a site where the periosteum developed into the lining of the otic capsule (Figure 3C). Strong immunoreactivity was present along the nerve fibers lying within the stroma and projected toward the sensory epithelia, as in week 8 of gestation (Figure 1F). All epithelial cells of the membranous labyrinth were void of immunoreactivity. A similar pattern was apparent in the adjacent cochlea (Figure 3C). Specific staining for S100 was also observed in the glia cells of Scarpa's ganglion as in prior stages (Figure 1G).

**Synaptophysin** expression also intensified at W11 in the utricle with intensely stained nerve fibers which projected into the developing macular organ. Expression intensified dramatically in the hair cells of the cristae and otolith sensory cells. This paralleled the slight extension of peripherin stained fibers which entered the base of the hair cells in the sensory epithelia of the cristae. The expression pattern of synaptophysin in the cochlea was marked by increased intensity of staining, with the positive fibers only reaching the epithelia of the middle turn (Pechriggl et al., 2015). In contrast, the synaptophysin staining in the vestibular end organs during W10 was marked by the complete staining of the nerve fibers as well as staining for the vestibular hair cells (Figure 3D).

Expression for the proliferation marker **Ki-67** was apparent at W11 with patchy staining in the otic epithelium (Figure 3E). Hair cells with distinct cilia bundles lined the sensory epithelia and exhibited immunoreactivity for this marker. These cells were likely terminally differentiated and must have completed their proliferation process. Many cells of the basal sensory epithelia were positive and possibly contained prosensory cells capable of proliferation and differentiation toward both cell types (Figure 3F). In the mesenchyme as well as the otic capsule, there was a more even distribution of positive cells without hot spots of cellular division.

### Sensory innervation and cell proliferation at W12

**Figure 4:**
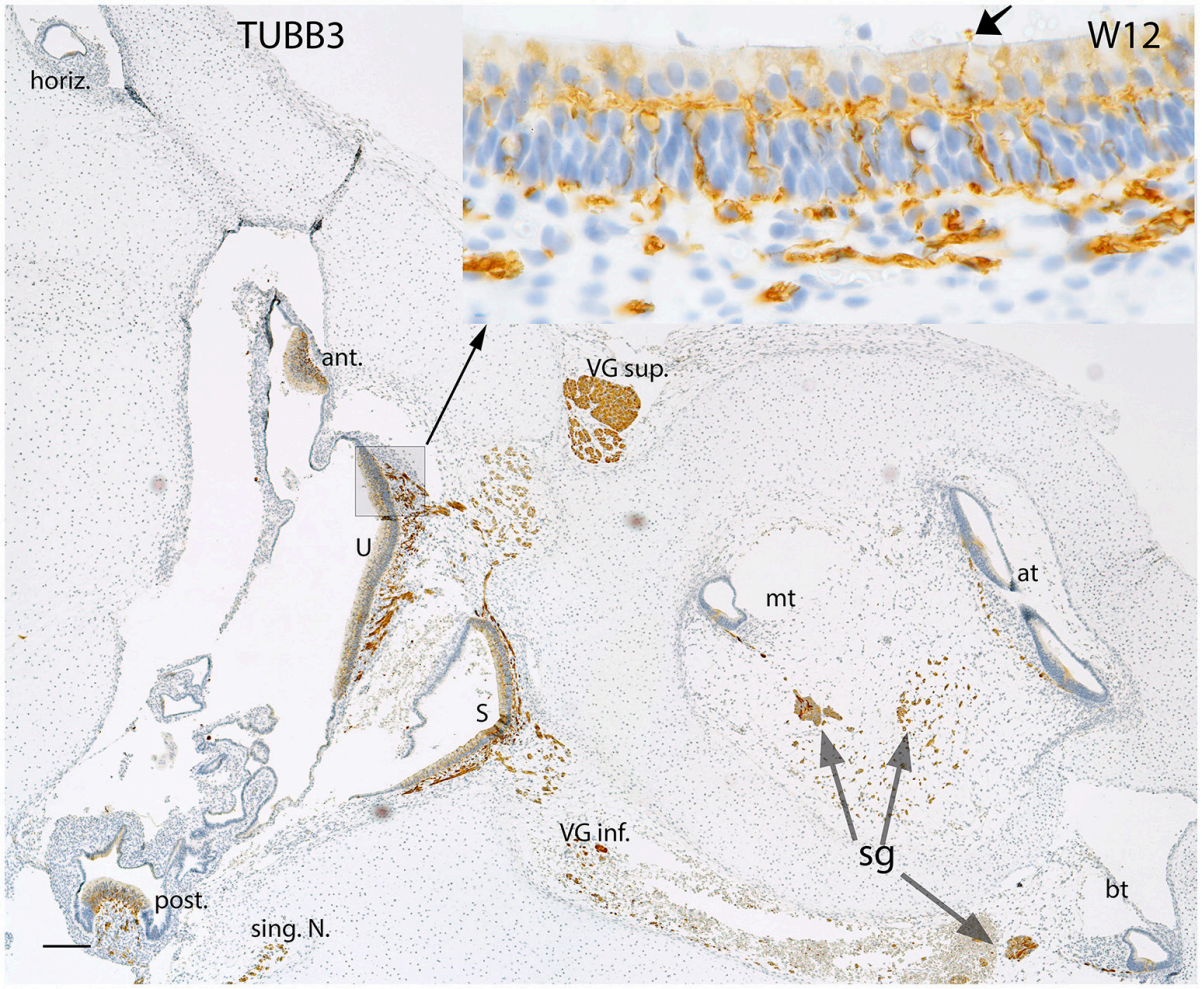
**Beta III tubulin (TUBB3) immunoreactivity in an overview image of the fetal inner ear at W12**. The immunoreactivity is apparent in the nerve fibers innervating the ampullary organs (ant, anterior; horiz, horizontal; post, posterior cristae) and the macular organs (U, utricle; S, saccule) of the vestibule. The singular nerve (sing.N) as well as the nerve fibers of both the vestibular ganglia (VG inf., Vestibular ganglion inferior; VG sup., Vestibular ganglion superior) are immunoreactive for Beta III tubulin. Inset exhibits the stained fibers intermingling with the base of the hair cells. The fibers extending from the base to apex of the future Organ of Corti are also positive for this marker. The nerve fibers are also apparent at the apical turn (at), middle turn (mt), and the basal turn (bt). The nerve fibers of the spiral ganglion (sg) distinctly express TUBB3. The somata of the sg also exhibit TUBB3. Scale bar: 50 μm.

Expression for the neuronal marker **Beta III tubulin** (Figure 4) displayed the progression of innervation in the vestibular end organs compared to the cochlea. Nerve fibers reached both the ampullar as well as the macular organs (Figure 4, inset) with intensely immunoreactive nerve fibers which sprouted onto the sensory epithelia of five end organs. Beta III tubulin better depicted the nerve fiber network underneath the hair cells, and some hair cells interestingly exhibited immunoreactivity in their cytoplasm, while some nerve fibers overshot into the endolymphatic compartment (Figure 4, inset arrow). The vestibular ganglia were also intensely stained by this marker. The immunoreactivity was also visible in the spiral ganglion of the cochlea at this stage with the stained fibers extending all the way from the base to the apex within the future OC.

### Pax genes activity in inner ear morphogenesis

**Figure 5:**
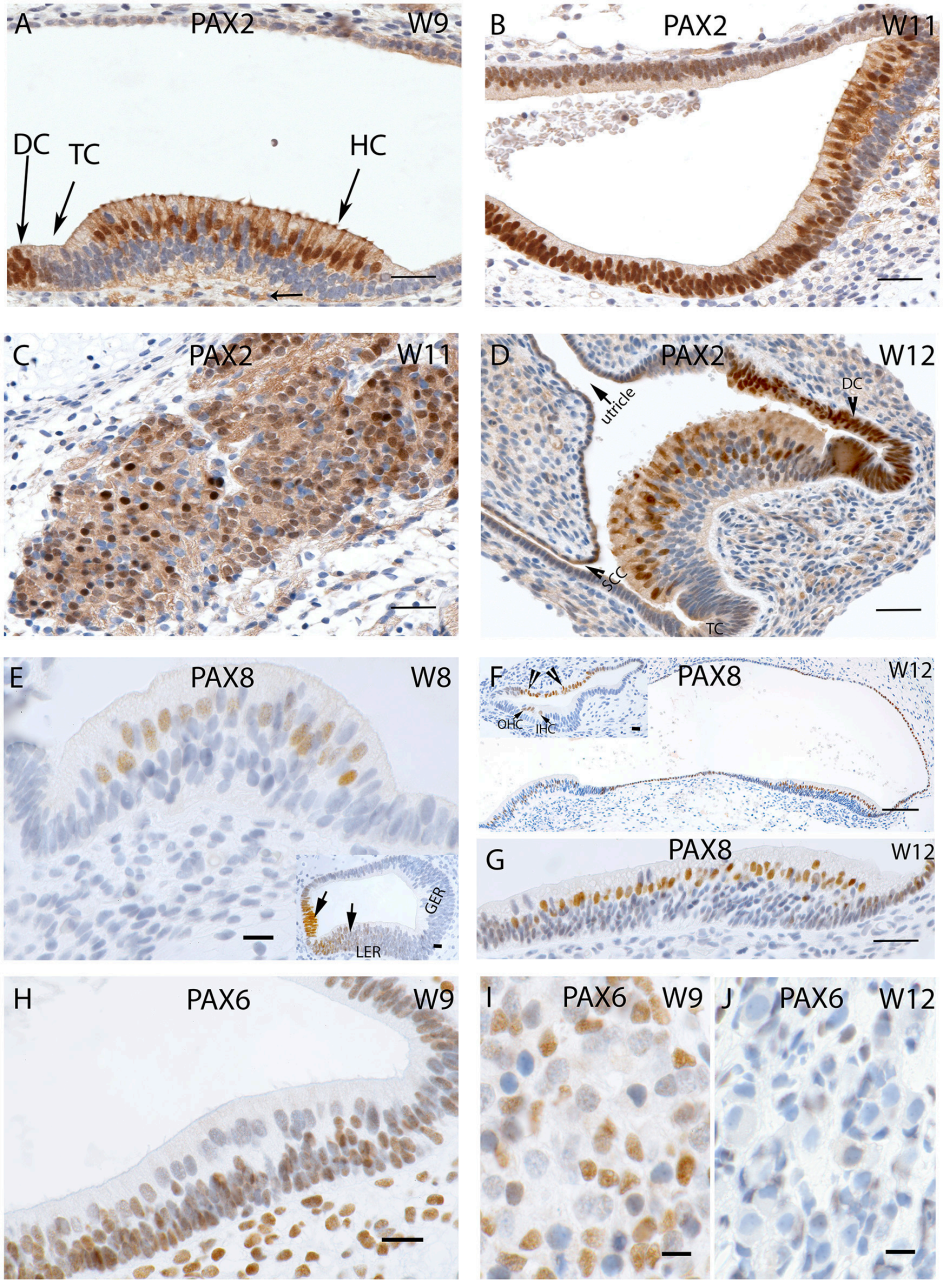
**(A)** PAX2 expression in a crista ampullaris at W9 with staining apparent in the nuclei and cytoplasm of the hair cells as well in the DC's **(B)** PAX2 exhibits intense cytoplasmic expression in the saccule and epithelial cells of the membranous labyrinth. **(C)** Intense and specific staining is apparent at the VG neurons at W11 with different intensities in cell nuclei. **(D)** PAX2 expression in an ampullary crest at W12, hair cells and the DC are positive for this marker. However, the TC is negative. There is a gradient of lower staining toward the periphery. The semi-circular canals side (SCC) (arrow head) shows less immunoreactivity compared the utricular side of the crest. **(E)** PAX8 immunostaining is visible in the otic epithelia among the future hair cells of the cristae ampullaris at W8. (**E**, inset) the staining for PAX8 is also visible in the developing cochlea where portions of the Lesser epithelial ridge (LER) and the future Organ of Corti are immunopositive for this marker. **(F)** PAX8 expression at W12 is confined to the hair cells of the ampullary crests and the utricle. More evident is the PAX8 immunoreactivity among the hair cells of the utricle **(G)** at higher magnification. (**F**, inset) image is the basal turn of the developing cochlea at W12 were clearly visible staining for PAX8 is evident in the Outer hair cells (OHC), Inner hair cells (IHC) as well as stria marginal cells and the Reissner's membrane (arrows). **(H)** PAX6 expression is visible in all epithelial as well as mesemchymal cells at W9. Only the differentiated hair cells are void of any immunoreactivity. **(I)** PAX6 immunoreactivity at W9 in the satellite glia and Schwann cells of the vestibular ganglion. **(J)** PAX6 immunoreactivity is no longer apparent at W12, satellite glial cells show a more adult-like morphology indicating advancement in the VG developmental process. Scale bar: 12 μm **(A–D,G)**, 50 μm **(F)**, 10 μm **(E)** 8 μm (**H–J** and insets of **E,F**).

Specific staining for the Pax2 antibody was observed in the nuclei and the cytoplasm of the developing hair cells and the dark cells of the ampullary organs at W9, while transitional zones were not reactive (Figure 5A). Less intense staining was apparent among the nerve fibers which innervated the cristae. A similar cytoplasmic staining pattern was visible in the saccule with strong staining among the hair cells as well as in the cells which line the otic epithelia (Figure 5B). Additional intense and specific staining for this antibody was observed in the Scarpa neurons during the same stage (Figure 5C), and negative nuclei within the ganglion were likely comprised of immature satellite glia or Schwann cells. Neurons exhibited immune reactivity for this transcription factor in the spiral ganglion of the fetal cochlea during this stage of development (Pechriggl et al., 2015).

Immunoreactivity for Pax2 was also prominent among the cell nuclei of the hair cells of the ampullae (Figure 5D) at W12. Only cells of the transitional and dark cell zone which faced the utricular aspect were positive, while the transitional cells (TC) and DC that merged into the canal side were negative. Keeping in mind that this transcription factor was expressed in the hair cells, the Lesser Epithelial Ridge (LER), and stria marginal cells together with cells of Reissner's membrane of the cochlea, we observed the corresponding expression in the utricular TC and DC zone and hair cells. The cell nuclei of the cuboidal epithelial cells of the membranous labyrinth were negative for Pax2. Pax2 staining was not restricted to nuclear expression; we found some less intense staining in the afferent nerve fibers innervating the end organs, and decent staining in the cytoplasm of the cuboidal epithelial cells separating the endolymph from perilymph.

Staining for the Pax8 was first apparent in the nuclei of the future hair cells of the otic epithelia of the ampulla at W8 (Figure 5E) as well as in the developing cochlea where the future cells of the stria vascularis were stained (Figure 5E, inset). We added the information of the cochlea here because there were no previous reports about this protein in humans. The LER exhibited staining, while the Greater Epithelial Ridge (GER) and part of the future Organ of Corti (where the hair cells are not differentiated at this stage) were void of staining at W8. Around W12, expression was restricted to the nuclei of the hair cells in the utricle and adjacent cuboidal epithelium, while these cells were void of immunoreactivity toward the anterior ampulla. In the ampullar organs, we found expression of this protein only in the cell nuclei hair cells and dark cells. No staining was found in the PSL cells or epithelia of the developing ampulla. In W12, Pax8 expression was confined to the hair cells of the ampullar and macular organs (Figure 5G) with the supporting cells remaining unstained in these end organs (Figure 5F). The simple cuboidal epithelia in between these end organs were also positive for this marker, while the TCs in between were negative. In the cochlea, three rows of OHC and one row of IHCs were positive at this stage, while the cells of the marginal cells of the stria vascularis were also positive for this marker (Figure 5F, inset). Immunopositive hair cells, dark cells, and marginal stria cells reflected corresponding structures in all sensory organs of the inner ear.

Staining for the nuclear marker Pax6 first showed up in the membranous labyrinth at W9. In the macular organs, otic epithelial cells as well as mesenchymal cells exhibited immunoreactivity (Figure 5H). Differentiated hair cells at this stage showed apical cilia and were already differentiated as indicated by their dearth in reactivity. Likewise, vestibular neurons were devoid of staining in contrast to the glia cells and surrounding mensenchyme (Figure 5I). This staining pattern disappeared by W12 of gestation (Figure 5J), when satellite glia cells changed to their final shape with a smaller and denser nucleus. None of the cells in the ganglion exhibited staining.

Absence of immunoreactivity for the apoptosis marker Caspase 3 was observed in all the vestibular end organs and in adjacent nerves between the gestational weeks 8 and 12 (data not shown). Summary of expression timeline of selected markers in the development of the human inner ear between 8 and 12 weeks of gestation is shown in Table 2.

**Table 2:** **Summary of the expression timeline of selected markers in the development of the human inner ear between 8 and 12 weeks of gestation**.

**Marker subtypes**	**Antibodies used**	**Week 8**	**Week 9–10**	**Week 11**	**Week 12**
Neuronal	Peripherin	Immunopositive nerve fibers in the sensory epithelia of vestibular end organs. Peripheral and central processes of the Scarpa ganglia positive	Equivalent expression as in W8	Immunopositive fibers enter base of the hair cells (HC) of the cristae	Equivalent expression as in W11
	Class III β-tubulin	Immunopositive nerve fibers innervate the vestibular end organs. Peripheral and central processes of the Scarpa's ganglia positive. Unequal development within the neuronal population	Increasing immunoreactivity		
Neurotransmission associated proteins	Synaptophysin	HC's, axons and cytoplasm of all neurons are positive. Widespread expression in the Scarpa ganglion neurons	Expression intensifies in the nerve fibers innervating the macular organs as well as in the HC's		
	Glutamine synthetase	Transitional cells (TC) of the utricle, supporting cells in the sensory epithelia satellite glia cells in the Scarpa's ganglia positive	Putative non-myelinated Schwann cells lying underneath the ampullary organs showed distinct immunoreactivity		Increasing immunoreactivity
Non-sensory cells	S100	Chondrocytes, mesenchymal tissue and nerve fibers projecting toward the sensory epithelia positive	Satellite glia cells and unmyelinated Schwann cells immunopositive	Increasing expression	
Cellular patterning	MAF B	Scarpa ganglion neuronal sub-population immunopositive	Equivalent expression as in W8		
	PAX2	HC's immunopositive with less intense expression in the nerve fibers innervating them		Intense expression in the Scarpa neurons	
	PAX8	HC's, cuboidal epithelial lining between the end organs immunopositive	Equivalent expression as in W8		Expression confined to the HC's of the ampullary crest and the utricle
	PAX6	Expression absent	Epithelial mesenchymal and glial cells exhibit immunoreactivity		Expression absent
Proliferation	Ki67	Planum semilunatum (PSL), dark cells, TC's adjacent to the cristae positive, simple cuboidal epithelial cells, mesenchyme and chondrocytes are also immunopositive	Declining expression	Patchy staining in the otic epithelium, HC's, mesenchyme as well as the basal part of the future supporting cells	Declining expression
Apoptosis	Caspase-3	Expression absent in all the weeks examined			

### Electron microscope images W12

**Figure 6:**
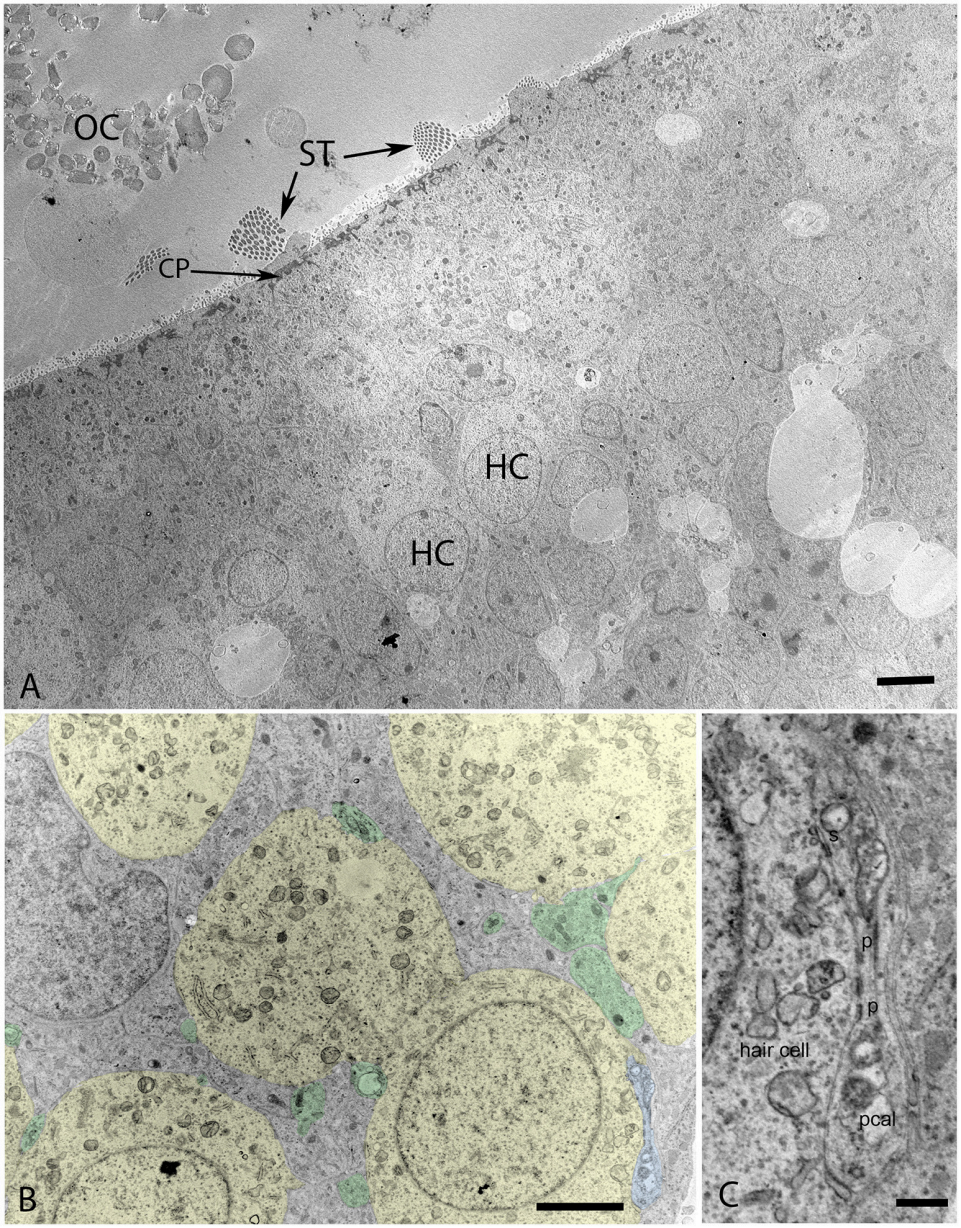
**Transmission electron micrograph of otolith organ (utricle) at W12 (A) with the hair cells (HC), stereocilia (ST), cuticular plate (CP), and immature otoconia (OC) visible. (B)** Identifies the formation of the protocalyx (colored blue) visible during this stage with the somata of the vestibular hair cells (colored yellow) and embedded between supporting cells (not colored), small presynaptic terminals and nerve fibers are highlighted in green. The higher magnified view of the protocalyx (pcal) in **(C)** reveals synaptic contacts (s) with vesicles at the presynaptic membrane and punctum adherens contacts (p) already formed between the protocalyx and the cell body of the hair cell. Various small and bigger vesicles in the hair cell cytosol can be seen in the vicinity of this protocalyx, indicating active transport mechanisms. Scale bar: 2 μm **(A,B)**, 500 nm **(C)**.

Transmission electron microscopy of specimens from the W12 showed clearly visible stereocilia (ST), with the kinocila not yet discernible at this stage. The terminal web composed of actin filaments called cuticular plates in hair cells was only weakly formed (Figure 6A). It underlies and anchors the stereocilia and appeared darkly stained. Hair cells possessed a less electron-dense cytoplasm with big nuclei rich in heterochromatin. Type I and II hair cells were not yet distinguishable from each other at this stage. The calyx synapses were not yet formed (Figures 6A,B), but numerous bouton contacts on the hair cells had attached to the hair cell somata (Figure 6B, colored green). We could not observe any synaptic ribbons indicating the immature state of synaptic contacts (Figure 6B). Nonetheless, some indications for proto-calyx formation were visible (Figure 6B, colored blue; Figure 6C). Differently sized otoconia filled the space apical to the hair bundles (Figure 6A). The higher magnified view of the protocalyx (pcal) in Figure 6C revealed synaptic contacts (s) with vesicles at the presynaptic membrane and punctum adherens contacts (p) already formed between the protocalyx and the cell body of the hair cell. Various small and bigger vesicles in the hair cell cytosol were visible in the vicinity of this protocalyx.

**Figure 7:**
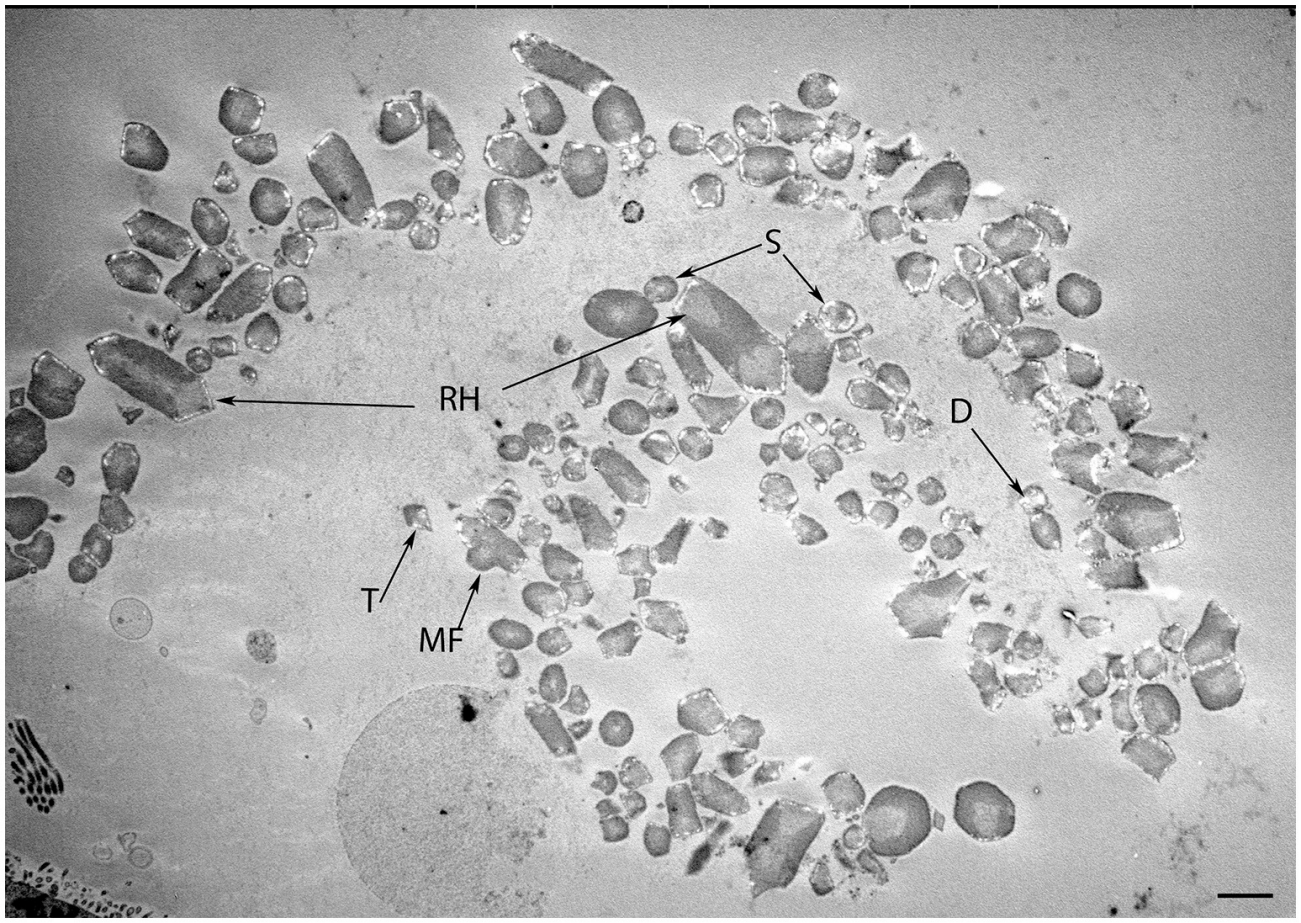
**Transmission micrograph image of the developing human fetal utricular otoconia in singlet (S), doublet (D), trigonal (T), multifaceted (MF), and rhombohedral (RH) shapes at W12 of gestation. Scale bar 10 μm**.

**Figure 8:**
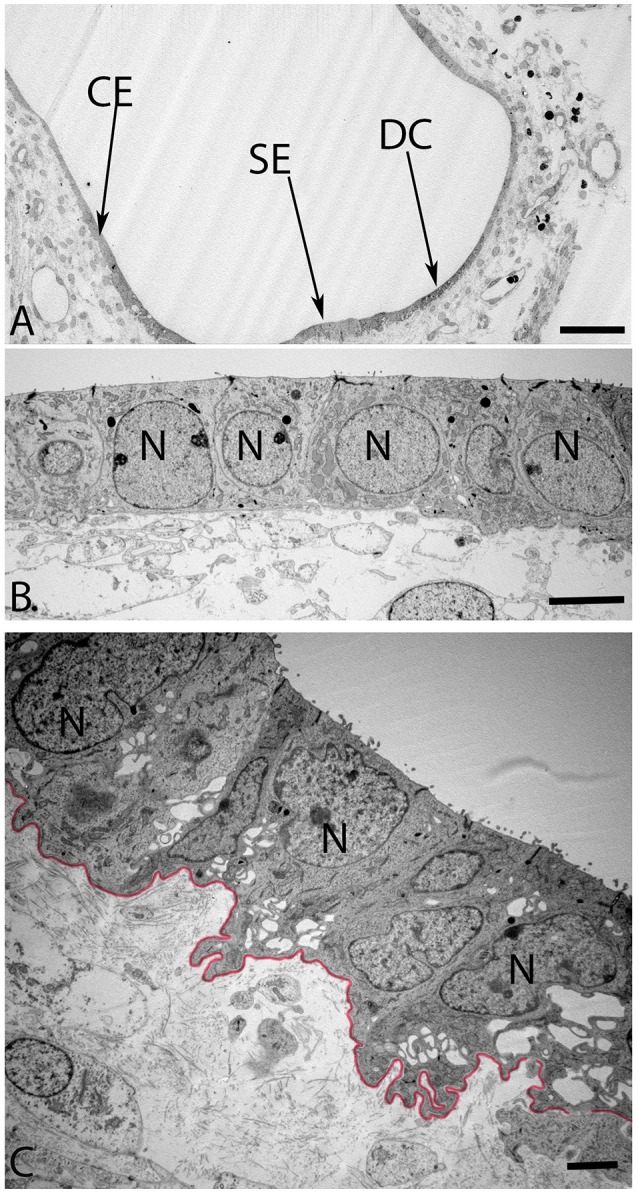
**Overview of the crista ampullaris in the human fetal VO at W12 in (A)**. The DC area of the cristae (arrow) is clearly distinct from both the lower portion of sensory epithelium (SE) close to the transitional zone (arrow) and the simple cuboidal epithelium (CE) (arrow). The cells in the CE **(B)** is single layered and uniformly sized while the DC **(C)** begin to develop their basal labyrinth with various spaces separated by a basal lamina (colored red) toward the mesothelial compartments. Large lobulated nuclei (N) placed in the upper portion of the cells further characterize these cells. Most of the cytoplasmic organelles are located in the upper two-thirds of the DC's, including numerous irregular vacuoles. Deep infoldings of the lateral and basal plasmalemma divide the lower third of these cells into numerous foliate cytoplasmic compartments containing mitochondria **(C)**. Scale bar: 20 μm **(A)**, 2 μm **(B)**, and 1 μm **(C)**.

The maturing otoconia were hexagonally shaped with a central dense core of otoconin protein being calcified by the addition of carbonate crystals (Figure 7). The calcification process was still not complete as revealed by the white spots among these crystals, which were the sites of initiation of the calcium deposition in its carbonate form. (Figure 8A) gives an overview of the sensory epithelium close to the transitional zone, dark cell area, and the simple cuboidal epithelium in a semi-circular canal. (Figure 8B) is a higher magnified view of the simple cuboidal epithelium and (Figure 8C) shows the DC area of the ampullary crest. The cells in the CE were uniformly shaped with a centrally placed nucleus, while those in the DC area had an irregular, laminated appearance with a large, lobulated nucleus. The nuclei of these cells were electron dense and the cytoplasm in these cells contained numerous irregular vacuoles. Deep infoldings of the lateral and basal plasmalemma divided the lower third of these cells into numerous cytoplasmic compartments containing mitochondria. This basal labyrinth was an indication for the extensive transport that these cells must accomplish, which necessitates an extended surface area. The basal lamina (Figure 8C, colored red) clearly delineated this epithelium from the underlying mesothelial tissue.

**Figure 9:**
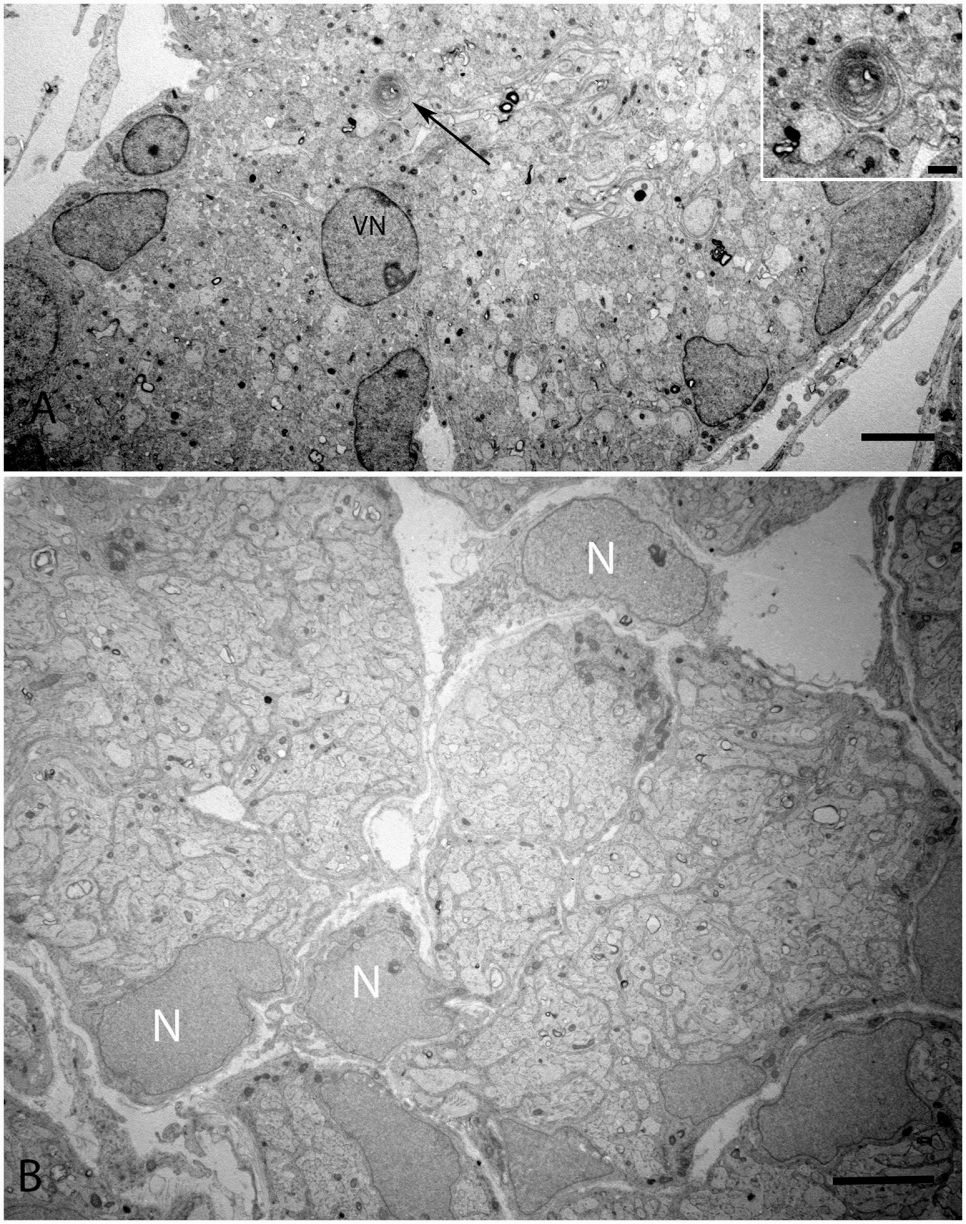
**Overview of the vestibular nerve close to the ganglion in the human fetal VO with the onset of myelin formation (arrow) visible in (A)**. Inset image exhibits the loose myelin structures. The vestibular neuron (VN) can clearly be distinguished by the nucleus structure, while other cell types possess high variability in cell nucleus structure. These cells surround nerve fibers, most of them are probably non-myelinating Schwann cells starting to wrap axons. **(B)** visualizes an adjacent portion of the vestibular nerve where the compartmentalization of the fascicles have already occurred with highly active nuclei (N) with no heterochromatin visible. Scale bar: 2 μm **(A,B)**, 500 nm (**A**, inset.)

The onset of the invagination of myelin sheaths was shown in (Figure 9A) while in (Figure 9B), the compartmentalization of the vestibular axons by sheaths of immature Schwann cells was complete. The myelination process had only started in few spots; the nuclei of these immature Schwann were void of any heterochromatin, revealing them to be highly active for their impending task of forming myelin sheets around each individual axon.

## Discussion

The assemblage of the neuronal circuitry as well its associated functional units requires the synchronous action of a large number of neuronal structural components, neurotransmitters, catalyzing enzymes and transcription factors. The progression of this developmental mechanism also necessitates its upregulation to be mediated by proliferation markers and its downregulation by apoptosis markers. It is this spatially and temporally controlled action during embryogenesis that shapes vestibular machinery as well as its nervous circuitry.

Awareness of the progression of expression of the factors involved in the development of the inner ear/VO is vital to better understanding inner ear disease etiologies as well as their progression. Previous developmental studies focused on murine inner ear models which were used to draw inferences, though the murine inner ear was not considered analogous to the human inner ear. These models need to be supplemented with human studies as the onset of vestibular function differs. Studies performed on neonatal rat pups have shown that the initiation of vestibular function, as demonstrated via the vestibular short latency evoked potentials (VsEPs), starts at postnatal day (PND) 8, while hearing as measured by auditory brain stem response (ABR) potentials were observed only at PND14 (Freeman et al., 1999). This indicates that although balance function emerges quite early in rats in comparison to hearing, it is still not a precise model to compare with the human VO developmental model. Studies were performed on newborn humans using vestibular evoked myogenic potential (VEMP) as an indicator of the development and maturation of the vestibular end organ. Studies showed that the sacculo-collic reflex which is responsible for head movements is already evoked by PND5 (Young et al., 2009). Patch clamp studies performed on human fetal vestibular hair cells and afferent calyxes have shown that major functional characteristics of both have emerged by GW18 (Lim et al., 2014).

Expression for the neuronal intermediate filament protein peripherin was found in the nerve fibers penetrating the sensorineural epithelium of the developing VO. The staining intensity for this neuronal filament increases with the progression in the gestational age and penetrates further into the sensory epithelia as the developmental age progresses. Previous immunostaining studies performed on the vestibular sensory organs from chinchilla, mice, and rats have shown that the staining for peripherin is confined to the extra-striolar regions of the utricle as well as to the peripheral portions of the crista ampullaris, with the immunoreactivity in the vestibular ganglia in these species being confined to the small vestibular ganglionic cells having fine axons (Lysakowski et al., 1999). In contrast, the peripherin-positive nerve fibers innervate the entire developing vestibular sensory organs of the human fetal utricle and cristae. Studies performed on adult gerbil specimens have shown immunoreactivity for peripherin in the vestibular ganglion as well as in the neuroepithelium of the cristae, with the staining being exclusive to the afferent neurons (Leonard and Kevetter, 2002). The expression of peripherin has been associated with the process of axonal extension and regeneration (Liu et al., 2009). In organotypic cultures performed on axotomized cochlear neurons, two-thirds of all the fibers were positive for peripherin (Lallemend et al., 2007), possibly implying that this neuronal marker could play a role in future regeneration models.

Immunostaining for synaptophysin is present in the nerve fibers innervating the sensory epithelia as well as in the afferent calyxes of the hair cells in the fetal VO, which in turn is of functional significance as it is an exceptional indicator of ongoing growth, synaptogenesis and neuronal activity in these afferent fibers surrounding the hair cells. This expression pattern parallels that observed in the mice embryo models (Scarfone et al., 1991). Previous research from our group could show the same progressive increase in staining in the fetal cochlea during the same time period in the GER region of the cochlea which gets remodeled into the future organ of Corti (Pechriggl et al., 2015).

The transcription factor Pax2 is necessary for the formation of the otic placode as observed in a Pax2 and Pax8^−/−^ mice (Bouchard et al., 2010), while mutations in Pax2 have been implicated in genetic malformations which could result in optic nerve malformation and hearing loss (McCarroll et al., 2012). Interestingly, we were able to detect this marker in the nuclei and cytoplasm of the vestibular hair cells toward W9, while in the fetal cochlea the same was observed at W11. Also, the expression of Pax8 was seen at W12 and was confined to the hair cells of the sensory organs of the fetal VO. Since Pax8 marker activity is correlated with Pax2, immunoreactivity for this marker is an indicator that the developmental process in the VO might still be ongoing during this stage of gestation.

Expression of the transcription factor Pax6, whose deletion has been found to result in aniridia, autism, and mental retardation in humans (Davis et al., 2008), was localized to the sensory epithelia at W9 among the supporting cells as well in the surrounding mesenchymal tissue. The role of Pax6 in the fetal VO is as yet uncertain due to the absence of supporting literature, though mutations in Pax6 have been found to cause cerebral deformities due to an undeveloped or absence of the anterior commissure and a decrease in the area of the corpus callosum (Bamiou et al., 2004b). Both of these affected regions have been found to contain auditory interhemispheric fibers resulting in substantial interhemispheric transfer deficits as well as hearing difficulties in patients with Pax6 mutations (Bamiou et al., 2004a), which might have an influence on auditory processing. Pax6, a regulator of cellular differentiation, could be regulating cellular proliferation at W9 with its expression falling toward W12, which leads to the hypothesis that the satellite glial cells and the Schwann cells are matured by W12.

Staining for S100 was confined to smaller cells traveling with the axons (Figure 1G). Also, the fact that immunoreactivity did not proceed through the basal lamina separating the sensory epithelium from underlying mesothelial connective tissue (Figure 1F) allowed us to suppose that these cells were unmyelinated Schwann cells. Likewise we identified mostly unmyelinated Schwann cells at electron microscopic level (compare Figure 9). Schwann cells did not enter the sensory epithelium since supporting cells acted as glia like cells for the innervating peripheral axons; the situation was likewise in the organ of Corti.

The specific expression of glutamine synthetase also supports the hypothesis that these sites of intense staining—the transitional zone as well as the developing satellite glial cells—are involved in glutamate detoxification in the fetal inner ear.

Previous research had shown the expression of this marker as early as the fifth week of gestation, with the expression decreasing toward the ninth week of gestation (Tafra et al., 2014). The expression of Ki-67 is apparent in the region of the macula and is restricted to the undifferentiated cells, whereas the differentiated hair cells with stereocilia are negative for this marker. Inactivity of the Caspase-3 in our study parallels the same previously observed in the human fetal cochlea (Pechriggl et al., 2015).

Expression for MAF B was found in a subpopulation of VG cells at W8. This subpopulation of cells was found to go down in number at W12. This neuronal marker which had earlier been identified to play an important role in the auditory circuit assembly (Lu et al., 2011) as well as in directing the differentiation process in the auditory neurons (Yu et al., 2013) is playing an as yet unidentified role, probably one as a driver of the synaptogenesis process among the neurons of the vestibular ganglion.

In our specimens, the formation of the protocalyx is apparent at the electron microscopic level at W12 and reveals synaptic contacts at the presynaptic membrane and the developing punctum adherens contacts between the protocalyx and the hair cell body. Patch-clamp studies on fetal vestibular calyxes have revealed that the developmental processes which ultimately lead to the formation of the type I and type II hair cells as well as the functional calyx terminals are completed by Week 18 (Lim et al., 2014). Previous work had shown that immature otoconia are still present at W14 (Dechesne and Sans, 1985), suggesting that the maturation process for these crystalline structures has paused down between these stages. Moreover, parallel to that which is seen in fetal rat inner ear specimens (Salamat et al., 1980), the calcium carbonate distribution in the rhombohedral (RH) otoconia was quite uneven, especially in the peripheral zones, as apparent from the varying distribution of electron density. This uneven distribution is indicative of the fact that the central core of organic matter in the otoconia is still seeding the calcite crystals in concert with the enzyme carbonic anhydrase (CA) during this stage of vestibular development. This observation correlates to the previous research (Yamashita et al., 1992) where strong expression for CA I and CA II in the endolymphatic sac of the developing human embryo during the gestational weeks 11 and 12 was observed.

## Author contributions

LJC: performed the immunostaining, collected data, analyzed the data, wrote the paper, EP: developed the concept, collected specimens, analyzed the data, HF: participated in data interpretation, revised manuscript, HR: participated in data interpretation, revised manuscript, MB: performed transmission electron microscopy, participated in revising the manuscript, AS: developed the concept, analyzed data, participated in data interpretation, revised manuscript, RG: developed the concept, participated in data interpretation, revised the manuscript. Both LJC and EP contributed equally toward this manuscript and should hence be considered as joint first authors of this manuscript.

### Conflict of interest statement

The authors declare that the research was conducted in the absence of any commercial or financial relationships that could be construed as a potential conflict of interest.

## Abbreviations

CE: Simple cuboidal epithelium
CNS: Central Nervous System
CP: Cuticular Plate
DC: Dark cells
GER: greater epithelial ridge
GLUT: glutamine
HC: Hair cells
IHC: inner hair cells
LER: lesser epithelial ridge
MAF B: V-maf musculoaponeurotic fibrosarcoma oncogene homolog B
MES: Mesenchyme
OHC: Outer hair cells
OtC: Otic Capsule
PAX: Paired box protein
Pe: Peripherin
PSL: Planum semilunatum layer
RTU: Ready to use
S: Saccule
ScG: Scarpa ganglion
SG: spiral ganglion
ST: Stereocillia
Sy: synaptophysin
TC: Transitional cells
U: Utricle
VO: Vestibular organ.
